# An increase in nitric oxide produced by rat peritoneal neutrophils is not involved in cell apoptosis

**DOI:** 10.1155/S0962935195000366

**Published:** 1995-05

**Authors:** I. M. Fierro, C. Barja-Fidalgo, R. M. Canedo, F. Q. Cunha, S. H. Ferreira

**Affiliations:** 1 Department of Pharmacology I.B. Universidade do Estado do Rio de Janeiro Ribeirão Preto 14049-900 Brazil; 2 Department of Pharmacology Faculty of Medicine of Ribeirão Preto Universidade de São Paulo Avenida Bandeirantes, 3900 Ribeirão Preto 14049-900 Brazil

## Abstract

Polymorphonuclear neutrophils (PMN) obtained from carrageenin-stimulated peritoneal cavities of rats, but not blood PMN, spontaneously produced nitric oxide (NO) when incubated *in vitro*. Incubation of the cells with the NO synthase inhibitors, L-imino-ethyl-L-ornithine (L-NIO) or N^G^-monomethyl-L-arginine (L-NMMA), inhibited NO production. This inhibition could be reversed by L-arginine. Incubation of PMN with lipopolysaccharide (LPS) failed to enhance NO production. Pretreatment of the rats with dexamethasone (DEXA) prior to carrageenin injection or incubation of PMN with the glucocorticoid *in vitro* partially inhibited the spontaneous release of NO. On the other hand, when PMN obtained from DEXA pretreated rats were incubated *in vitro* with DEXA, NO synthase activity and hence NO generation were almost abolished. A similar inhibition was also observed following the addition of L-NIO or cycloheximide to cultures of carrageenin-elicited PMN. The NO production by PMN did not appear to be related to cell viability or apoptosis. Indeed, neither the blockade of NO generation by L-NIO nor the incubation of the neutrophils with a NO donor, S-nitroso-acetylpenicillamine (SNAP) modified the pattern of LDH release or DNA fragmentation. In summary, it appears that PMN migration triggers a continuous NO synthesis, and that NO produced by these cells is not related to their apoptosis.


Mediators of Inflammation 4, 209–216 (1995)

### Analysis of the IFN-γ-induced signal transduction pathway in fetal rejection

Irene Athanassakis

Department of Biology, University of Crete, P.O. Box 1470, Heraklion 711-10, Crete, Greece

**The placenta, one of the most important fetal tissues during gestation, ensures nutrition, development and protection of the fetus. Although placenta lacks expression of class II MHC antigens, they can be induced either by interferon-gamma (IFN-γ) on the spongiotrophoblast zone, or by 5-azacytidine (5-azaC) on the labyrinthine trophoblast zone, two agents actively participating in a plethora of immunological and inflammatory reactions. This induction is correlated with fetal abortion and fetal developmental abnormalities. In this work the *in vitro* and *in vivo* signal transduction pathways followed by IFN-γ or 5-azaC to induce class II antigen expression on placental cells by using specific pathway inhibitors has been studied. It is shown that at least three intracellular pathways are implicated in the Ia induction. $p21^{ras}$ is the first protein activated by the two agents while further signalling requires $Ca^{2+}$ mobilization and PKC activations. When the *in vitro* results are transferred to live animals using the same inducing agents and pathway inhibitors, it is found that theophylline ($Ca^{2+}$/CaM inhibitor) and anti-$p21^{ras}$ are the most potent suppressors of the IFN-γ- and 5-azaC-induced side effects during pregnancy. The data presented here point to novel directions not only as to the intracellular signalling, but also to the use of pathway inhibitors *in vivo* to treat aberrant antigen expression associated with fetal loss.**

**Key words:** 5-AzaC, Class II antigens, Fetal abortion, IFN-γ, Placenta, Signal transduction.

### Introduction

Cellular crosstalk is the fundamental basis of development, differentiation and survival of any pluricellular organism. Protein binding to a cell membrane receptor initiates intracellular signalling reactions leading to the regulation of any physiological process. The principal architecture of the signal transduction pathways known up to now, includes specific receptors, GTP-binding proteins, second messenger generating protein kinases, target functional proteins and regulatory proteins.

This study is an analysis of the signal transduction pathway initiated by IFN-γ leading to class II major histocompatibility complex (MHC) antigen expression on the adherent placental cells, which previous studies have identified as spongiotrophoblasts.[1] IFN-γ is known to induce class II antigens in different human and murine cells and tissues, including leukocytes, monocytes, glioblastomas and astrocytes.[1–9] However, the intracellular pathway followed each time depends on the species and the cell type studied. In humans, IFN-γ activates the $Ca^{2+}$/Calmodulin ($Ca^{2+}$/CaM) pathway in most cases.[5,8] The second messenger calcium ions bind to calmodulin, triggering conformational changes in this protein, and allowing the activation of many enzymes, including protein kinases.[10] This $Ca^{2+}$/CaM pathway can be blocked by inhibitors like theophylline and W7 (see the Methods section and Reference 5).

In the murine system, the induction of class II antigens by IFN-γ also involves the protein kinase C (PKC) pathway.[6–8] Stimulation of receptors or opening of $Ca^{2+}$ channels initiates hydrolysis of phosphatidyl inositol (PtdIns) by phospholipase C where the subsequently produced diacylglycerol (DAG) activates PKC, thus providing a linkage between extracellular and intracellular responses. Although DAG is the most important stimulator of PKC, the latter can also be regulated by other phospholipid derived messengers.[11] Upon activation of PKC, multiple positive and negative regulatory signals may affect a ple-

© 1995 Rapid Communications of Oxford Ltd thora of target functional proteins. A way of studying the events occurring downstream of PKC activation is by applying specific inhibitors, such as sphingosine and staurosporine.[6,8]

Another important system shown in some cases to be involved in class II antigen induction by IFN-γ in humans as well as in mice, is the G-protein system.[9,12] It consists of a complicated cascade of GTP-binding proteins located in the inner cell membrane, able to cross-link many cell surface receptors to effector proteins and initiate many intracellular events.[12] This pathway was approached by applying mevalonic acid lactone (MEV) to the cells which, although it does not have well defined properties,[13,14] is employed as a G-protein (ras) suppressor.[8]

Study of the signal transduction mechanisms leading to class II antigen expression on murine trophoblasts are very important because these cells directly regulate the feto-placental unit development and control whether the outcome of pregnancy is successful or not. Placental trophoblasts have been found not to express class II MHC antigens[1,15,16] and this negative state is one of the protective mechanisms evoked by the fetus to escape maternal immune attack and avoid autoimmune reactions. It has been shown previously in mice that the two trophoblast layers of the placenta differ in their inducibility to express class II antigens. Spongiotrophoblast derived trophoblasts are found to be under regulatory control and are susceptible to induction of class II antigens by IFN-γ *in vitro* and *in vivo*.[8,17,18] Trophoblasts in the inner placental layer (labyrinthine trophoblast zone) can only be induced to express these antigens upon stimulation with the demethylating agent 5-azaC *in vitro* as well as *in vivo*.[8,19] In both systems studied, class II antigen induction is preceded by the activation of $p21^{ras}$.[20]

In the present communication, a possible signal transduction pathway is proposed, triggered by IFN-γ and 5-azaC leading to class II antigen and $p21^{ras}$ expression on placental adherent cells. It is shown that class II antigen induction by both agents requires activation of $Ca^{2+}$/CaM, PKC and $p21^{ras}$. Intracellular signalling begins with $p21^{ras}$ activation since only MEV inhibits induction of $p21^{ras}$ and class II antigen expression. This step is followed by $Ca^{2+}$/CaM and PKC activation, whereas the pathway of tyrosine kinase (TK) is not involved in this process. The importance of these results lies in *in vivo* experiments where an attempt was made to reverse the IFN-γ- and 5-azaC-induced harmful effects by injecting the treated pregnant mice with specific pathway inhibitors. The results show that appropriate treatments can rescue fetal abortion and in most cases (depending on the inhibitor used) lead the feto–placental development to physiological levels.

### Materials and Methods

*Mice:* BALB/cJ and C3H/HeJ mice were maintained in our Animal Breeding Unit at the University of Crete (Greece). Each BALB/cJ or C3H/HeJ female was individually caged with a BALB/cJ or C3H/HeJ male overnight and the following morning the females were examined for the presence of a vaginal plug. The morning where the plug was observed was considered as day zero of pregnancy.

*Pathway inhibitors:* Theophylline (TPH) and W7 (*N*-(6-aminohexyl)-5-chloro-1-napthalenesulfonamide HCl) were used to block the $Ca^{2+}$/CaM pathway.[5] TPH blocks the interaction between CaM and enzymes (inactive adenyl cyclase). This drug is able to block the class II MHC antigen induction by IFN-γ on the human cell lines HL-60, HeLa, U937, a Burkitt's lymphoma, and Daudi cells.[5] From the literature, dose–response experiments place the best working concentration at 0.15 mg/ml. For this work and cellular system (the trophoblast portion of adherent placental cells), doses of 0.2 and 0.1 mg/ml (Sigma, St Louis, MO) were applied. The higher dose (0.2 mg/ml) gave the best results as to the reduction of class II MHC antigen expression by IFN-γ and 5-azaC. W7 is a CaM antagonist that binds CaM and inhibits calcium ion–CaM regulated activities. In the cellular systems used by Ina *et al.*[5], dose–response experiments (10, 20, 30 μM) showed that the best working conditions are obtained with 10 and 20 μM of W7. For the present system, W7 (Sigma) was used *in vitro* at the concentration of 15 μM, which gave the best responses.

Sphingosine (SPH) and staurosporine (St) are potent inhibitors of the PKC pathway, which are known to inhibit the activity of PKC. SPH has been used in several cell lines to block the class II antigen induction by IFN-γ, including human THP-1, murine WEHI-3, bone marrow macrophages, where dose–response experiments (1, 10, 50 μM) have shown that 50 μM offers maximal inhibition.[6,8] In the present work 50 μM and 25 μM of SPH (Sigma) were applied to the adherent placental cells and it was found that the best reduction of class II antigen expression induced by IFN-γ and 5-azaC is obtained at the concentration of 50 μM (15 μg/ml). St inhibits the phospholipid calcium ion dependent protein kinase C. This product was purchased from UBI (Lake Placid, NY) and showed the best inhibitory effect on the induction of class II antigen expression by IFN-γ and 5-azaC at the concentration of 50 nM, which is in agreement with the dose–response experiments performed by Gumina *et al.*[6]

Mevalonic acid (MEV) is an intermediate product in cholesterol synthesis and it is claimed to interfere with the G protein pathway through HMG–CoA and farnesylation of cysteins.[12] MEV was purchased from Sigma and tested at the concentrations of 5 mM (0.65 mg/ml) and 50 mM (6.5 mg/ml). Since both concentrations effectively inhibited class II and $p21^{ras}$ expression induced by IFN-γ and 5-azaC with no apparent toxic effects, the lowest concentration was used.

Genistein (Gen, 4′,5,7-trihydroxyisoflavone), a potent inhibitor of tyrosine-specific protein kinase (TK),[9] was purchased from UBI and tested at concentrations of 10, 30 and 50 μg/ml. None of these concentrations was able to block the class II or $p21^{ras}$ antigen expression induced by IFN-γ and 5-azaC. However, this drug when applied to a tyrosine kinase dependent system (T98G human glioblastoma) shows its inhibitory activity even at the dose of 10 μg/ml.

*Antibodies:* Rat anti-$p21^{ras}$ neutralizing monoclonal antibody (Ab-1, Oncogene Sc. Inc., Manhasset, NY), mouse anti-$IA^d$ or $IA^k$ or $K^d$ or $K^k$ monoclonal antibodies (Becton Dickinson, Meshelen, Belgium), mouse monoclonal anti-cytokeratin antibody (PKK1, Labsystems, Helsinki, Finland), mouse monoclonal anti-vimentin antibody (PK-V, Labsystems) and rat anti-mouse Mac-1 antibody (Caltag Lab, San Francisco, CA) were used at a concentration of 1 μg/ml for *in vitro* detection of the equivalent proteins. Anti-mouse or anti-rat IgG coupled to FITC antibodies (Sigma) were used at a dilution of 1/100 and 1/150, respectively. Mouse IgG or rat IgG were used as negative controls to the above antibodies.

*Placental cell cultures:* Placental cell cultures were performed as described previously[21] with slight modifications. Briefly, placentae were isolated from BALB/cJ × BALB/cJ or BALB/cJ × C3H/HeJ pregnant mice on day 11 to 14 of gestation and, after removing the maternally derived decidual cap layer, the tissue was cut into small pieces and single cell suspensions were prepared by passing the preparation through a syringe with a 18½G needle, into Hank's solution.[1] The cells were washed three times and cultured at the concentration of $1 \times 10^6$ cells/ml in RPMI 1640 medium supplemented with 10% fetal calf serum (FCS, Seralab, Sussex, England) in 35 mm Falcon plates (Becton Dickinson, Oxnard, CA). On the fourth day of culture the cells were washed to eliminate the non-adherent cells and growth factor containing medium, which was then replaced with fresh RPMI 10% FCS medium, and the next day IFN-γ (100 U/ml, Holland Biotechnology, Leiden, The Netherlands) or 5-azaC (2 μM, Sigma) with or without the various inhibitors were added to the cells. All cultures were incubated at 37°C, 5% $CO_2$ until the seventh day of culture, where marker analysis experiments by the immunofluorescence technique were performed on the adherent layer of cells.

In vivo *treatment of pregnant females:* BALB/cJ females pregnant with BALB/cJ males were injected intraperitoneally from day 6 to 11 of gestation with 5000 U recombinant IFN-γ (0.5 μg/mouse) per day, or 5-azaC (10 μg/injection), or phosphate buffered solution (PBS, Gibco, Grand Island, NY). The IFN-γ- or 5-azaC-treated animals were additionally injected with the TPH and SPH pathway inhibitors or antibodies to $p21^{ras}$ and class II antigens. TPH was administered i.p. at the dose of 0.15 mg/injection to pregnant mice on days 6 and 10 of gestation, whereas SPH was given i.p. at the dose of 15 μg/injection on days 6, 8 and 10 of pregnancy. These doses were determined from preliminary experiments by injecting various doses of TPH and SPH from day 6 to 11 of gestation. Once the lowest effective dose was estimated, the time intervals between the injections was increased to finally determine the doses described above. The neutralizing anti-class II antibody was injected i.v. at a dose of 50 μg/injection on days 8 and 10 of pregnancy. The neutralizing anti-$p21^{ras}$ was injected *in vivo* i.p. into pregnant mice at the concentration of 5 μg/injection on days 6, 7, 9 and 10 of gestation. Higher doses of this antibody given i.v. or i.p. at longer time intervals (20–30 μg on days 8, 10 or 11) significantly affected fetal viability (data not shown). On the 12th day of gestation the animals were killed by cervical dislocation and fetal resorptions were evaluated. Placentae and fetuses were isolated and weighed using a high precision balance (Sartorius 2434).

*Indirect immunofluorescence:* Immunofluorescent staining was performed as described previously.[1] Briefly, adherent placental cells were lifted from the culture plates using cell scrapers, washed with PBS supplemented with 0.2% bovine serum albumin (BSA, Sigma) and 0.01% sodium azide (Sigma, PBS–BSA–azide) and placed in 96-well V-bottomed plates at a concentration of $1 \times 10^6$ cells/well. For cytoplasmic protein detection, the cells were permeabilized with ice-cold methanol (20% for 20 min at 4°C). Following this step (where necessary) the cells

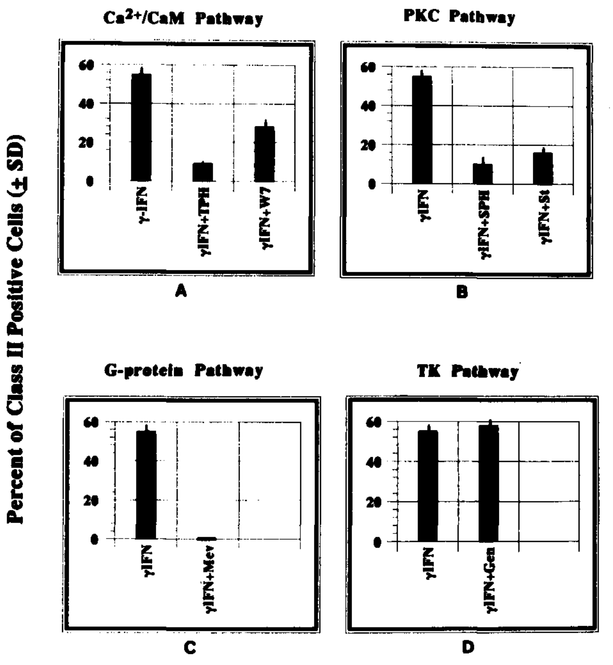

FIG. 1. Net modification of IFN-γ-induced class II antigen expression on adherent placental cells by different pathway inhibitors. Primary adherent placental cells were treated on day 5 with IFN-γ ± inhibitors and tested for class II antigen expression (using monoclonal anti-IA$^d$ or anti-IA$^k$ antibodies) on the seventh day by immunofluorescence (see Methods). TPH (theophylline) and W7 were used as inhibitors for the $Ca^{2+}$/CaM pathway, SPH (sphingosine) and St (staurosporine) as inhibitors for the PKC pathway, Mev (mevalonic acid) for the G-protein pathway and Gen (genistein) for the TK pathway ($n=5$). Doses are given in Methods. Vertical lines represent the standard deviation (SD) of triplicate counts.

*Note 1:* Net values represent the subtracted difference between the specific antibody reacted cells and negative control, which did not exceed 10%.

*Note 2:* The experiments in this study were conducted on unfractionated adherent placental cells, having the following characteristics: cytokeratin, 64 ± 1%; vimentin, 2 ± 1%; Mac-1, 65 ± 1%; class I, 65 ± 1%; class II, 3 ± 1%, as assessed by immunofluorescence staining.

were reacted first with 1% normal goat serum, then with the test antibody and finally with a FITC conjugated anti-mouse or anti-rat antibody. All incubations were performed at 4°C for 30 min. Extensive washing followed each step of the procedure. The cells were immobilized with 25% glycerol, mounted on slides and fluorescence intensity was evaluated using a Zeiss (Oberkochen, Germany) fluorescence microscope. Cells with weak or no staining were scored as negative. Positive cells were considered as those showing bright to very bright fluorescence intensity. The results concerning p21$^{ras}$ and class II MHC antigen regulation (shown in the figures) are expressed as the net percentage of fluorescent cells (test group – negative control group), ± standard deviation.

*Statistical analysis:* In all experiments, Student's *t*-test was used for the evaluation of significance levels ($p$). All other statistical values show the mean ± standard deviation (S.D.).

### Results

In vitro *study of signal transduction pathways leading to class II and p21$^{ras}$ expression by IFN-γ:* Previous studies have shown that IFN-γ is a potent inducer of class II MHC antigens on the spongiotrophoblasts.[1] In this work, the intracellular pathways stimulated by IFN-γ, leading to class II antigen expression are analysed, and the involvement of four different pathways, $Ca^{2+}$/CaM, PKC, G-proteins and TK, is tested. Primary placental cells were prepared as described in the Methods section and on the fifth day of culture these were stimulated with IFN-γ and various inhibitors. The presence of TPH or W7 in these IFN-γ-stimulated placental cell cultures resulted in a 85 and 50% ($p < 0.001$ and $p < 0.005$) reduction of class II antigen expression, respectively, as tested by immunofluorescence (Fig. 1(A)). Although both inhibitors block the $Ca^{2+}$/CaM pathway, their action is localized at different levels (see Methods), which may explain the difference in the degree of inhibition. These results indicate that class II antigen induction by IFN-γ requires mobilization of $Ca^{2+}$ (see Discussion).

In order to assess intracellular cross-talk with other pathways, placental cell cultures were treated with SPH and staurosporine, two potent inhibitors of PKC activation. These agents caused 82 and 71% inhibition ($p < 0.001$) of class II expression, respectively, as compared with IFN-γ-induced cells (Fig. 1(B)). Thus, PKC is also involved in the IFN-γ signal transduction pathway leading to class II antigen expression.

It has been previously reported that the expression of p21$^{ras}$ is stimulated in the IFN-γ-treated trophoblast cells.[20] In order to further test whether intracellular cross-talk with the G-protein pathway is necessary to IFN-γ-induced class II antigen expression, MEV was applied to the adherent placental cells. The application of this agent completely suppressed class II antigen induction, giving only background staining of the cells (Fig. 1(C)). MEV was unable to suppress IFN-γ-induced expression of class II antigens on human HL-60 and HeLa cells as well as murine WEHI-3 and 70Z cell lines (data not shown). Finally, the involvement of TK in this system was excluded, since application of genistein to the cultures did not have any effect on class II antigen expression (Fig. 1(D)).

Following the same reasoning and experimental protocols, the signal transduction pathway

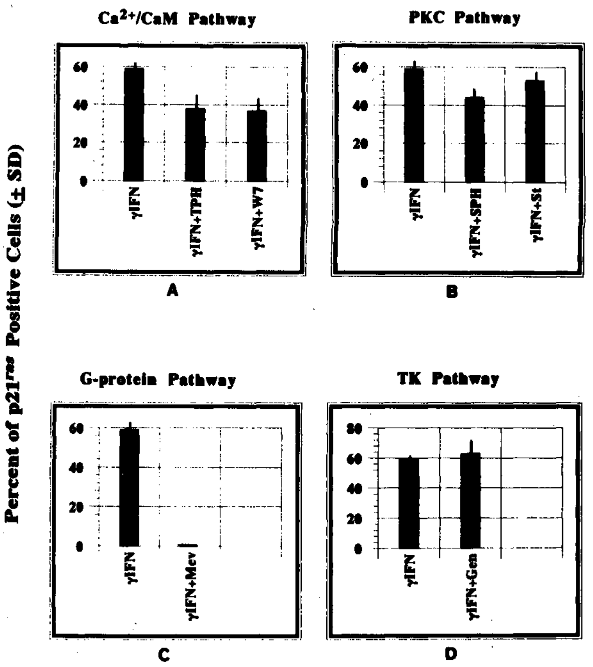

FIG. 2. Net modification of IFN-γ-induced p21*ras* expression on adherent placental cells by different pathway inhibitors. Primary adherent placental cells were treated on day 5 with IFN-γ ± inhibitors and tested for p21*ras* expression on the seventh day by immunofluorescence ($n = 5$, see Methods, and legend to Fig. 1). Vertical lines represent the standard deviation (S.D.) of triplicate counts. Net values and cell characterization are as given in Fig. 1.

leading to p21*ras* antigen expression after IFN-γ administration was studied. The results show that only mevalonic acid lactone completely ablated p21*ras* expression, whereas all the other inhibitors did not significantly affect this expression (Fig. 2). These results indicate that p21*ras* is an intermediate product to the class II antigen induction by IFN-γ, and its activation precedes the $Ca^{2+}$ mobilization and PKC activation.

In vivo *study of signal transduction pathways leading to class II and p21*ras *expression by IFN-γ:* It has been shown previously by this laboratory that administration of IFN-γ to pregnant mice from day 6 to 11 of gestation increases fetal abortion, reduces fetal size and, among other dysfunctions observed at the maternal level, it increases the size of the spleen approximately 3-fold.[8] These IFN-γ-induced events were associated with the expression of class II MHC antigens as well as the p21*ras* protein on the spongiotrophoblast layer of the placenta.

In order to test whether the *in vitro* results described above can also be applied *in vivo*, pregnant mice were injected with IFN-γ and inhibitors (doses and timing given in Methods). On the 12th day of pregnancy, the mice were sacrificed and the weight of placenta, fetus and maternal spleen as well as the percentage of fetal abortion was recorded: TPH and SPH, when administered during the IFN-γ treatment, reduce fetal abortion to normal levels, whereas IFN-γ treatment alone increases the percentage of fetal loss 3-fold, as compared with untreated controls (Table 1). However, only TPH significantly, but not completely, corrects the effect of IFN-γ in fetal and spleen weight (Table 1). Thus, TPH causes an 88% increase in fetal weight ($p < 0.001$), and a 46% reduction in spleen weight ($p < 0.005$), as compared with IFN-γ treatment alone. SPH gives a 38% reduction ($p < 0.005$) in spleen weight but it does not significantly increase fetal size (Table 1). Placental weight is not affected in either case. While testing the toxicity of TPH and SPH during pregnancy, control experiments show that the doses used do not affect fetal viability or maternal spleen weight, but reduce fetal weight non-significantly.

It was then attempted to confirm, in an additional way, the above described results by injecting the IFN-γ-treated pregnant mice with neutralizing monoclonal antibodies against p21*ras* and class II antigenic determinants. The combination of IFN-γ + anti-p21*ras* gives a 51% and 123% increase in placental and fetal weight respectively, as compared with the IFN-γ treatment alone. Spleen weight is reduced by 41% ($p < 0.005$), whereas the percentage of fetal abortion returns to physiological levels (Table 1). The combination IFN-γ + anti-Ia reduced fetal abortion to normal levels and increased placental and fetal weight by 43% and 100% respectively, as compared with IFN-γ-treated pregnant mice. However, this treatment did not significantly reduce the maternal spleen weight (Table 1, see Discussion).

In vitro *and* in vivo *study of signal transduction pathways leading to class II and p21*ras *expression by 5-azaC:* It is known that the demethylating agent 5-azaC induces class II and p21*ras* antigen expression on trophoblasts derived from the labyrinthine trophoblast zone of the placenta.[1,20] It is shown that class II antigen expression is affected by the same inhibitors as for the IFN-γ-induced expression of these antigens. Specifically, TPH and W7 reduce the 5-azaC-included class II antigen expression by 54% and 38% respectively ($p < 0.005$), as compared with 5-azaC treated cells (Fig. 3(A)). Furthermore, SPH and St inhibit the expression of these antigens by 75% and 97% ($p < 0.001$) respectively (Fig. 3(B)), whereas the application of MEV gives 100% inhibition of Ia antigen expression (Fig. 3(C)). The TK pathway does not seem to

Table 1. Protection of fetal development from IFN-γ-induced side effects by specific pathway inhibitors *in vivo*

| Treatment* | Weight (mg ± S.D.) | | | Fetal resorption | |
|---|---|---|---|---|---|
| | Placenta** | Fetus** | Spleen | % | *n* |
| - | 94 ± 6 | 159 ± 8 | 118 ± 16 | 18 ± 3 | 10 |
| IFN-γ | 61 ± 6 | 43 ± 12 | 315 ± 20 | 54 ± 8 | 20 |
| IFN-γ + TPH | 85 ± 9 | 81 ± 9 | 169 ± 32 | 15 ± 1 | 8 |
| IFN-γ + SPH | 70 ± 4 | 57 ± 4 | 194 ± 2 | 15 ± 5 | 8 |
| IFN-γ + anti-p21$^{ras}$ | 92 ± 5 | 96 ± 11 | 185 ± 10 | 17 ± 5 | 10 |
| IFN-γ + anti-class II | 87 ± 9 | 86 ± 4 | 274 ± 21 | 15 ± 5 | 15 |
| TPH | 67 ± 9 | 64 ± 2 | 102 ± 20 | 25 ± 7 | 5 |
| SPH | 78 ± 15 | 72 ± 11 | 115 ± 16 | 15 ± 5 | 5 |
| anti-p21$^{ras}$ | 89 ± 5 | 99 ± 8 | 180 ± 10 | 0 | 8 |
| anti-class II | 91 ± 17 | 109 ± 9 | 124 ± 15 | 7 ± 3 | 8 |

*Pregnant mice were treated as described in Methods. Briefly, IFN-γ (5 000 U/injection) was administered i.p. to the animals from day 6 to 11 of gestation. TPH (0.15 mg/injection) was given i.p. either alone or with IFN-γ on days 6 and 10 of pregnancy. SPH (15 μg/injection) was given i.p., alone or with IFN-γ on days 6, 8 and 10 of pregnancy. Anti-p21$^{ras}$ (5 μg/injection) was administered i.p., either alone or with IFN-γ on days 6, 7, 9 and 10 of gestation. Finally, anti-class II monoclonal antibody (anti-IA$^{d}$, 50 μg/injection) was administered i.v., either alone or with IFN-γ on days 8 and 10 of gestation. *p* values are given in the text.
**Resorbed sites were not included in the means.

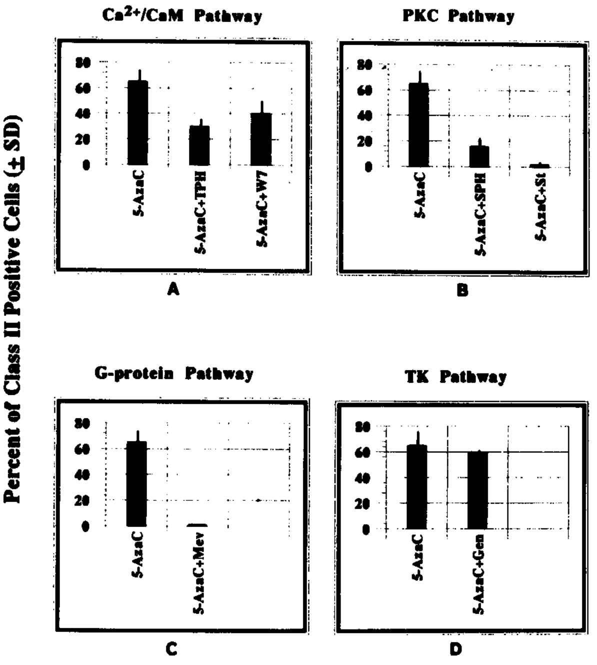

FIG. 3. Net modification of 5-azaC-induced class II antigen expression on adherent placental cells by different pathway inhibitors. Primary adherent placental cells were treated on day 5 with 5-azaC ± inhibitors and tested for class II antigen expression (using monoclonal anti-IA$^{d}$ or anti-IA$^{k}$ antibodies) on the seventh day by immunofluorescence ($n$ = 5, see Methods and legend to Fig. 1). Vertical lines represent the standard deviation (S.D.) of triplicate counts. Net values and cell characterization are as given in Fig. 1.

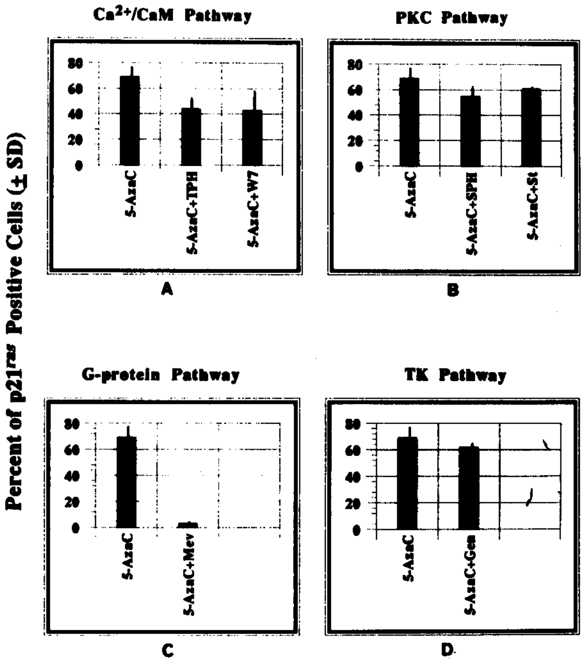

FIG. 4. Net modification of 5-azaC-induced p21$^{ras}$ expression on adherent placental cells by different pathway inhibitors. Primary adherent placental cells were treated on day 5 with 5-azaC ± inhibitors tested for p21$^{ras}$ expression on the seventh day by immunofluorescence ($n$ = 5, see Methods and legend to Fig. 1). Vertical lines represent the standard deviation (S.D.) of triplicate counts. Net values and cell characterization are as given in Fig. 1.

be involved in this system, since genistein does not affect the 5-azaC-induced expression of these antigens on placental cells (Fig. 3(D)). All these inhibitors were used at the same doses as those described for IFN-γ.

Application of these inhibitors in order to examine changes at the p21$^{ras}$ expression levels, showed that only MEV could block the appearance of the p21$^{ras}$ proteins (Fig. 4). Administration of 10 μg of 5-azaC to pregnant mice i.p. from days 6 to 11 of gestation, results either in complete fetal abortion or in very high percentages of fetal loss, where the surviving embryos (10 – 15%) have abnormal development.[19] Based on the *in vitro* results described above, the reversal of 5-azaC-induced events by TPH or

Table 2. Protection of fetal development from 5-azaC-induced side effects by specific pathway inhibitors *in vivo*

| Treatment* | Weight (mg ± S.D.) | | Fetal resorption | |
|---|---|---|---|---|
| | Placenta** | Fetus** | % | *n* |
| - | 94 ± 6 | 159 ± 8 | 18 ± 3 | 10 |
| 5-azaC | 52 ± 10 | 105 ± 18 | (100) | 20 |
| 5-azaC + TPH | 92 ± 15 | 97 ± 11 | 39 ± 5 | 8 |
| 5-azaC + SPH | 74 ± 6 | 90 ± 4 | 21 ± 9 | 8 |
| 5-azaC + anti-p21$^{ras}$ | 78 ± 12 | 88 ± 8 | 16 ± 4 | 12 |
| 5-azaC + anti-class II | 80 ± 7 | 115 ± 9 | 0 | 15 |
| TPH | 67 ± 9 | 64 ± 2 | 25 ± 7 | 5 |
| SPH | 78 ± 15 | 72 ± 11 | 15 ± 5 | 5 |
| anti-p21$^{ras}$ | 89 ± 5 | 99 ± 8 | 0 | 8 |
| anti-class II | 91 ± 17 | 109 ± 9 | 7 ± 3 | 8 |

*Pregnant mice were treated as described in Methods. Briefly, 5-azaC (10 µg/injection) was administered i.p. to the animals from day 6 to 11 of gestation. TPH (0.15 mg/injection) was given i.p., either alone or with 5-azaC on days 6 and 10 of pregnancy. SPH (15 µg/injection) was given i.p., either alone or with 5-azaC on days 6, 8 and 10 of pregnancy. Anti-p21$^{ras}$ (5 µg/injection) was administered i.p., either alone or with 5-azaC on days 6, 7, 9 and 10 of gestation. Finally, anti-class II monoclonal antibody (anti-IA$^{d}$, 50 µg/injection) was administered i.v., either alone or with 5-azaC on days 8 and 10 of gestation. *p* values are given in the text.
**Resorbed sites were not included in the means.

SPH, or anti-p21$^{ras}$ or anti-class II monoclonal antibodies, was attempted. All treatments rescued fetal abortion induced by 5-azaC alone and increased placental size to normal (Table 2). Fetal size was not significantly different from that in the untreated animals and spleen weight was not affected in any case (data not shown).

### Discussion

When a physiological or chemical agent comes in contact with the surface of a cell, second messenger signals are transmitted not only to downstream functional proteins, but also to upstream compounds and members of other signal transduction pathways in order to give this cell specific instructions about its function(s) and phenotype. The major event studied here is the induction of class II MHC antigens by IFN-γ on the placenta. Although the mechanisms of induction of these antigens by IFN-γ have been examined in many systems, including different cell types of different species, my interest in trophoblasts comes from the biological significance of this mechanism and the role it may play in escaping fetal abortion and ameliorating conditions of fetal survival. The results show that at least three pathways are involved in the signal transduction mechanism of class II antigen expression on trophoblasts, including $Ca^{2+}$ mobilization, PKC and p21$^{ras}$ activation. In cases such as pregnancy, graft implantation and autoimmune disorders, the expression of class II antigens has harmful effects on the organism and such activation has to be depressed. This is in contrast with systems studied by other investigators, where MHC class II induction benefits the organism by stimulating the immune system. Thus, after using adherent placental cells, the *in vitro* findings concerning inhibition of class II antigens via the three described pathways were transferred to *in vivo* experiments. It was shown that the inhibitors which block the induction of these antigens protect the fetus from abortion and ameliorate the conditions of fetal development.

In this study specific inhibitors of four different pathways were employed: TPH and W7 to suppress the $Ca^{2+}$/CaM system; SPH and staurosporine to inhibit PKC activation; mevalonic acid to block G-proteins (*ras*); and genistein to inhibit the TK system. All but genistein were able to block class II antigen expression induced by IFN-γ, on adherent placental cells.

It has been shown previously that the induction of class II antigens on trophoblasts is closely linked to the cellular *ras* oncogene, which was also found to be stimulated by the IFN-γ treatment on the same cells.[20] Using mevalonic acid lactone as an inhibitor of p21$^{ras}$, and consequently of G-proteins,[8] it was possible to block the class II antigen induction by IFN-γ. Results indicated that p21$^{ras}$ activation occurs upstream to the stimulation of class II antigens.

In order to place p21$^{ras}$ activation within the signal transduction pathway of IFN-γ and/or examine possible degrees of intracellular cross-talk, the inhibitors to $Ca^{2+}$/CaM, PKC and TK were tested for their ability to block the expression of p21$^{ras}$. With the exception of mevalonate, none of the inhibitors was able to inhibit such induction, indicating that p21$^{ras}$ activation is the first step in the signal transduction pathway leading to class II antigen expression.

It has been reported from this and other laboratories that administration of IFN-γ to pregnant mice affects feto–placental development as well as maternal physiology.[18,20,22] In view of the present *in vitro* results, I attempted to block the

*in vivo* IFN-γ-induced side effects by specific pathway inhibitors (TPH and SPH), anti-class II or anti-p21$^{ras}$ antibodies. All these treatments rescued the IFN-γ-induced abortion, TPH and anti-p21$^{ras}$ being the most efficient.

In the last section of this work, I concentrated on the mechanisms inducing class II antigen expression on the labyrinthine trophoblast after 5-azaC treatment. It is demonstrated that the same inhibitors blocking the IFN-γ-induced class II and p21$^{ras}$ expression, also inhibit the 5-azaC-induced stimulation, both *in vitro* and *in vivo*.

It is thus proposed that the sequence of events leading to class II expression and ultimately fetal rejection after IFN-γ or 5-azaC administration begins with an initial p21$^{ras}$ activation followed by either $Ca^{2+}$ mobilization and PKC activation or vice versa. This does not exclude the possibility that the two agents may follow or share other means of propagating their signals leading, however, to the same endpoint. *In vivo* application of pathway inhibitors is able to reverse the side effects not only at the feto–placental but also the maternal level. It is premature to speculate that the use of TPH, anti-p21$^{ras}$, SPH and to a lesser degree anti-class II antibodies, can be used as therapeutic agents. However, further experimentation may prove that administration of these or analogue reagents may have a potential role in the future. It is clear that aberrant expression of class II antigens on the murine placenta lead to fetal rejection.[1,18,20] These findings can be generalized to the role of IFN-γ-mediated class II upregulation in the induction of immune responses, graft rejection or autoimmune diseases. Although in the present study the class II inducers were given exogenously, multiple mechanisms including viral or microbial infection, cytokine overproduction, and hormonal disequilibrium, may lead to the production of IFN-γ and/or other inflammatory cytokines with synergistic potential like TNFα, IL-6, IL-1 and IL-12. All possibilities are valid until definitive proof can be provided, a task that is being undertaken at this laboratory.

### References

1. Athanassakis-Vassiliadis I, Thanos D, Papamatheakis J. Induction of class II major histocompatibility complex antigens in murine placenta by 5-azacytidine and interferon-γ involves different cell populations. *Eur J Immunol* 1989; **19:** 2341–2348.
2. Sher MG, Beller DI, Unanue ER. Demonstration of a soluble mediator that induces exudates rich in Ia-positive macrophages. *J Exp Med* 1980; **152:** 1684–1698.
3. Steeg PG, Moore RN, Johnson HM, Oppenheim JJ. Regulation of murine macrophage Ia antigen expression by a lymphokine with immune interferon activity. *J Exp Med* 1982; **156:** 1780–1793.
4. Zarling JM. Effects of interferon and its inducers on leucocytes and their immunologic functions. In: Came PE, Carter WA, eds., *Interferons and their Applications.* The Netherlands: Springer Verlag 1984; 403–431.
5. Ina Y, Koide Y, Nezu N, Yoshida TO. Regulation of HLA class II antigen expression: intracellular signaling molecules responsible for the regulation by γ-IFN and cross-linking of Fc receptors in HL-60 cells. *J Immunol* 1987; **139:** 1711–1717.
6. Gumina RJ, Freire-Moar J, DeYoung L, Webb DR, Devens BH. Transduction of the γ-IFN signal for HLA-DR expression in the promonocytic line THP-1 involves a late-acting PKC activity. *Cell Immunol* 1991; **138:** 265–279.
7. Benveniste EN, Vidovic M, Panek RB, Norris JG, Reddy AT, Benos DJ. Interferon-γ-induced astrocyte class II major histocompatibility complex gene expression is associated with both protein kinase C activation and $Na^+$ entry. *J Biol Chem* 1991; **266:** 18119–18126.
8. Vassiliadis S, Kyrpides N, Stravopodis D, Athanassakis I, Papamatheakis J. Investigation of intracellular signals generated by γ-IFN and IL-4 leading to the induction of class II antigen expression. *Med Inflamm* 1993; **2:** 343–348.
9. Ryu K, Koide Y, Yamashita Y, Yoshida TO. Inhibition of tyrosine phosphorylation prevents IFN-γ-induced HLA-DR molecule expression. *J Immunol* 1993; **150:** 1253–1262.
10. Cohen, P. Signal integration at the level of protein kinases, protein phosphatases and their substrates. *TIBS* 1992; **17:** 408–413.
11. Liscovitch M. Crosstalk among multiple signal-activated phospholipases. *TIBS* 1992; **17:** 393–399.
12. Helper JR, Gilman AG. G proteins. *TIBS* 1992; **17:** 383–387.
13. Schafer WR, Kim R, Sterne R, Thorner J, Kim SH, Rine J. Genetic and pharmacological suppression of oncogenic mutations in Ras genes of yeast and humans. *Science* 1989; **245:** 379–385.
14. Roullet JB, Xue H, Pappu AS, Roullet C, Holcomb S, McCarron DA. Mevalonate availability and cardiovascular functions. *Proc Natl Acad Sci USA* 1993; **90:** 11728–11732.
15. Chatterjee-Hasrouni S, Lala PK. MHC antigens on mouse trophoblast cells: paucity of Ia antigens despite the presence of H-2K and D. *J Immunol* 1981; **127:** 2070–2076.
16. Raghupathy R, Singh B, Leigh JB, Wegmann TG. The ontogeny and turnover kinetics on paternal H-2K antigenic determinants on the allogeneic murine placenta. *J Immunol* 1981; **127:** 2074–2079.
17. Vassiliadis S, Athanassakis I. Type II interferon may be a potential hazardous therapeutic agent during pregnancy. *Brit J Haematol* 1992; **82:** 782–783.
18. Vassiliadis S, Tsoukatos D, Athanassakis I. Interferon-induced class II expression at the spongiotrophoblast zone of the murine placenta is linked to fetal rejection and developmental abnormalities. *Acta Physiol Scand* 1994; **151:** 485–495.
19. Athanassakis-Vassiliadis I, Galanopoulos VK, Grigoriou M, Papamatheakis J. Induction of class II MHC antigen expression on murine placenta by 5-AzaC correlates with fetal abortion. *Cell Immunol* 1990; **128:** 438–449.
20. Athanassakis-Vassiliadis I, Vassiliadis S, Papamatheakis J. Common mechanisms goven the expression of p21$^{ras}$ and class II MHC antigens in the murine placenta. *J Reprod Immunol* 1992; **21:** 149–161.
21. Athanassakis I, Bleackley RC, Paetkau V, Guilbert L, Barr PJ, Wegmann TG. The immunostimulatory effect of T cells and T cell lymphokines on murine fetally-derived placental cells. *J Immunol* 1987; **138:** 37–44.
22. Chaouat G, Athanassakis I. Induction of abortion by Ds RNAs results in an abnormal MHC class I expression on murine labyrinthine trophoblast as well as the expression of detectable class II MHC antigens on placental spongiotrophoblasts. *EOS-J Immunol Immunopharmacol* 1992; **12:** 79.

ACKNOWLEDGMENTS. I thank Dr Simon Vassiliadis for helpful discussions and reviewing this manuscript. I also thank D. Tsoukatos and S. Kourletakis for technical assistance. This work was supported by University of Crete funds.

**Received 30 January 1995; accepted in revised form 16 March 1995**
